# Hemispheric lateralization of white matter microstructure in children and its potential role in sensory processing dysfunction

**DOI:** 10.3389/fnins.2023.1088052

**Published:** 2023-04-17

**Authors:** Shalin A. Parekh, Jamie Wren-Jarvis, Maia Lazerwitz, Mikaela A. Rowe, Rachel Powers, Ioanna Bourla, Lanya T. Cai, Robyn Chu, Kaitlyn Trimarchi, Rafael Garcia, Elysa J. Marco, Pratik Mukherjee

**Affiliations:** ^1^Department of Radiology and Biomedical Imaging, University of California–San Francisco, San Francisco, CA, United States; ^2^Cortica Healthcare, San Rafael, CA, United States; ^3^Department of Psychology and Neuroscience, University of Colorado Boulder, Boulder, CO, United States

**Keywords:** sensory processing disorder, lateralization, sensory over-responsivity, neurite orientation dispersion and density imaging, microstructure

## Abstract

Diffusion tensor imaging (DTI) studies have demonstrated white matter microstructural differences between the left and right hemispheres of the brain. However, the basis of these hemispheric asymmetries is not yet understood in terms of the biophysical properties of white matter microstructure, especially in children. There are reports of altered hemispheric white matter lateralization in ASD; however, this has not been studied in other related neurodevelopmental disorders such as sensory processing disorder (SPD). Firstly, we postulate that biophysical compartment modeling of diffusion MRI (dMRI), such as Neurite Orientation Dispersion and Density Imaging (NODDI), can elucidate the hemispheric microstructural asymmetries observed from DTI in children with neurodevelopmental concerns. Secondly, we hypothesize that sensory over-responsivity (SOR), a common type of SPD, will show altered hemispheric lateralization relative to children without SOR. Eighty-seven children (29 females, 58 males), ages 8–12 years, presenting at a community-based neurodevelopmental clinic were enrolled, 48 with SOR and 39 without. Participants were evaluated using the Sensory Processing 3 Dimensions (SP3D). Whole brain 3 T multi-shell multiband dMRI (*b* = 0, 1,000, 2,500 s/mm^2^) was performed. Tract Based Spatial Statistics were used to extract DTI and NODDI metrics from 20 bilateral tracts of the Johns Hopkins University White-Matter Tractography Atlas and the lateralization Index (LI) was calculated for each left–right tract pair. With DTI metrics, 12 of 20 tracts were left lateralized for fractional anisotropy and 17/20 tracts were right lateralized for axial diffusivity. These hemispheric asymmetries could be explained by NODDI metrics, including neurite density index (18/20 tracts left lateralized), orientation dispersion index (15/20 tracts left lateralized) and free water fraction (16/20 tracts lateralized). Children with SOR served as a test case of the utility of studying LI in neurodevelopmental disorders. Our data demonstrated increased lateralization in several tracts for both DTI and NODDI metrics in children with SOR, which were distinct for males versus females, when compared to children without SOR. Biophysical properties from NODDI can explain the hemispheric lateralization of white matter microstructure in children. As a patient-specific ratio, the lateralization index can eliminate scanner-related and inter-individual sources of variability and thus potentially serve as a clinically useful imaging biomarker for neurodevelopmental disorders.

## Introduction

Diffusion tensor imaging (DTI) studies have shown inherent microstructural asymmetries in the human brain (Ocklenburg et al., 2016). White matter tracts such as the cingulum bundle, posterior limb of the internal capsule, corticospinal tract, superior cerebellar peduncle and arcuate fasciculus are known to be left lateralized while tracts like the inferior longitudinal fasciculus, parts of the superior longitudinal fasciculus, the anterior limb of the internal capsule and the uncinate fasciculus have been shown to be right lateralized on diffusion anisotropy in the human brain (Park et al., 2004; Gong et al., 2005; Yasmin et al., 2009; Takao et al., 2011; Madsen et al., 2012; Takao et al., 2013; DeRosse et al., 2015). It is believed that such hemispheric asymmetries reflect the functional lateralization of the human brain. The most well-known example of functional lateralization is that of language to the left cerebral hemisphere and visuospatial processing to the right cerebral hemisphere (Gotts et al., 2013).

Altered hemispheric lateralization on DTI has been seen in neurodevelopmental disorders like autistic spectrum disorder (ASD), schizophrenia and dyslexia (Fletcher et al., 2010; Lange et al., 2010; Lo et al., 2011; Ocklenburg et al., 2016; Postema et al., 2019; Andica et al., 2021; Floris et al., 2021). While a number of studies have shown loss of normal left lateralization in large association tracts and temporal lobes in autistic children and adolescents (Lo et al., 2011) some studies have shown increased left lateralization (Leisman et al., 2022). The variation in results is likely due to heterogeneity in the autism group with different subjects showing differing symptoms and having different underlying etiologies. For example, Floris et al. (2021) showed that language delay in autistic children corelated with a rightward pattern while core autism symptoms severity correlated with a leftward pattern. A recent study has shown that it is difficult to discriminate imaging biomarkers of autism due to group and individual heterogeneity of phenotypic components or symptoms of autism (Xu et al., 2021). Due to this heterogeneity, it is important to study the individual phenotypic components that make up this heterogeneity in neurodevelopmental disorders (Chang et al., 2014). One such phenotypic component is sensory dysfunction (Chang et al., 2014). Children with autism have difficulties in sensory processing, which is now considered a diagnostic hallmark of autism. As per the Diagnostic and Statistical Manual-5 (DSM-5) hyper- and hypo-reactivity to sensory input (characteristic of sensory modulation) is a core criterion for ASD. This has now resulted in interest in learning the pathophysiological basis of sensory processing and sensory dysfunction and changes in brain microstructure associated with sensory processing dysfunction.

Sensory processing dysfunction (SPD) is difficulty in interpreting the sensory world in an adaptive way. There are different types of SPD including sensory modulation disorder, sensory discrimination disorder and sensory motor control disorder (Miller et al., 2012). These often coexist with each other. Sensory modulation is a core criterion in ASD. Sensory modulation can be further categorized as sensory over responsivity (SOR), sensory under responsivity and sensory craving (Miller et al., 2012). Sensory over responsivity is manifest as exaggerated response to normal sensory stimuli with ordinary sensory stimulation leading to distress (Marco et al., 2011). These sensory stimuli can be auditory tactile or visual. Hemispheric lateralization has not yet been explored in children with SPD.

Brain microstructure has been studied using diffusion MRI (Ocklenburg et al., 2016). Traditional DTI is sensitive to changes in tissue microstructure but lacks specificity for individual tissue microstructural features. More recently, non-gaussian models with biophysical compartment modeling of brain microstructure are being used. One such model, Neurite Orientation Dispersion and Density Imaging (NODDI), enables the quantification of the intracellular volume fraction, also known as the neurite density index (NDI), as well as the axonal fiber orientation dispersion index (ODI) and the free water fraction (FISO). These compartment models are both sensitive and specific and provide more biologically meaningful measures of white matter microstructure than traditional DTI metrics, including for childhood white matter development (Chang et al., 2015) and for many neurological disorders such as traumatic brain injury (Palacios et al., 2020). The NODDI model has been shown to bear excellent correlation to brain microstructure on pathology specimens (Zhang et al., 2012). In adults with autism, the NODDI model has shown better accuracy and specificity than standard diffusion tensor metrics (Andica et al., 2021). There are limited studies of brain microstructural lateralization using the NODDI model in children. A recent study in adults found substantial asymmetries in gray matter microstructure using NODDI reflecting histopathological asymmetries. There were no effects of sex or handedness on these asymmetries (Schmitz et al., 2019). The excellent sensitivity and specificity of the NODDI model would also allow for in-depth understanding of potential signatures of white matter microstructural lateralization differences in children with SOR versus other forms of SPD.

Here, we evaluate lateralization of white matter microstructure using NODDI and conventional DTI parameters in children with neurodevelopmental concerns (NDC) between 8 and 12 years of age. First, we characterize white matter tract hemispheric lateralization in school age children with NDC using NODDI compared to DTI, postulating that the more biophysically meaningful NODDI metrics will help explain the direction of asymmetry observed from the DTI metrics. Then, we directly compare white matter tract hemispheric asymmetry for those children with versus without sensory over-responsivity. Children with and without SOR serve as a test case in the study of lateralization for neurodevelopmental disorders. Our exploratory hypothesis is that children with SOR will have altered regional hemispheric lateralization in white matter microstructure relative to children without SOR (non-SOR) using both DTI and NODDI.

## Methods

We prospectively enrolled children, ages 8–12 years, who presented at a community-based neurodevelopmental clinic (Cortica San Rafael, California) using a research protocol approved by the institutional review board at our medical center with written informed consent obtained from the parents or legal guardians and assent obtained from the study participants. Each study participant was recruited following medical intake at Cortica Healthcare. Each participant underwent a thorough review of their history, a general and neurodevelopmental physical examination, and record review by their physician and research coordinator. Children were screened for eligibility in part through the ESSENCE-Q-REV, a 12-question caregiver screener for ESSENCE disorders, including ASD, attention deficit hyperactivity disorder (ADHD), developmental coordination disorder, specific language impairment, and Tourette’s syndrome (Gillberg, 2010). The response options were “No,” “Maybe/A Little,” or “Yes.” The threshold for inclusion (“optimal cutoff”) for this measure was at least one ‘Yes’ or at least two ‘Maybe / A Little’ responses in total. Participants were excluded if they had (A) Nonverbal Index ≤70 on the Wechsler Intelligence Scale for Children (Fifth Edition) (Wechsler, 2014) (B) Below ESSENCE-Q-REV “optimal cutoff” for neurodevelopmental concerns (C) Caregiver(s) unable to complete intake forms (D) History of *in utero* toxin exposure (E) Gestational age < 32 weeks or intrauterine growth restriction (birth weight < 1,500 grams) (F) Hearing or visual impairment (G) Additional medical/neurologic condition, including active epilepsy, malignancy, or known brain injury/malformation (H) If they were found to have a research designation of ASD. Participants with ASD were excluded to obtain a more homogenous group so that microstructural changes on brain imaging would be reflective of sensory over-responsivity and not ASD. Participants scoring ≥15 on the Social Communication Questionnaire (Rutter et al., 2003) and above the diagnostic cut-off on the Autism Diagnostic Observation Schedule (Second Edition) (Lord et al., 2012) were considered to have a research designation of ASD.

Participants underwent direct sensory characterization through the Sensory Processing 3 Dimensions (SP3D) (Mulligan et al., 2019) scale, led by a licensed pediatric occupational therapist. Three auditory, four tactile, and three visual probes from the SP3D scale were utilized in the SOR cohort assignment process. Within each of these probes, a participant is given a score of 1 (typical), 2 (mild/moderate), or 3 (severe) in reference to the intensity of their aversive reaction. A score of 2 or 3 in any of the probes corresponds to an SOR designation in the respective domain(s), and therefore categorizes the participant into the SOR cohort. We did not exclude subjects who had other forms of sensory dysfunction like sensory under-responsivity or sensory modulation disorder and as such the non-SOR group may have subjects with these forms of sensory dysfunction.

All subjects were imaged on a single Siemens 3 Tesla (3 T) Prisma MRI scanner (Erlangen, Germany) using a 64-channel head coil. Participants were acclimated and desensitized to the MRI scanner environment (Horowitz-Kraus et al., 2016). Briefly, the child explored the scanner environment, and sat on the bed until comfortable. Head motions were minimized using foam padding on either side of the head-coil apparatus used for the scan. The child was positioned within the scanner bore and began watching a movie *via* an MRI-compatible audiovisual system, and image acquisition began. Communication was established between the child and study coordinator through headphones equipped with a built-in microphone. Verbal communication and positive reinforcement were maintained with the child throughout the scan. Scanning was terminated immediately if the child did not wish to continue. All children were awake during the entire duration of the scan.

Structural MRI of the brain was acquired with an axial 3D magnetization prepared rapid acquisition gradient-echo (MPRAGE) T1-weighted sequence. Whole brain diffusion MRI was performed at diffusion-weighting strengths (shells) of *b* = 1,000 s/mm^2^ (64 diffusion-encoding directions) and 2,500 s/mm^2^ (96 diffusion-encoding directions), with 5 *b* = 0 s/mm^2^ volumes per shell (TE = 72.20 ms, TR = 2,420 ms, flip angle = 85 degrees, slice thickness = 2.0 mm, matrix size = 220×110, FOV = 220 mm) using single-shot spin echo echoplanar imaging with forward and reverse phase encoding. Simultaneous multiband (MB) excitation was used (MB factor = 3). The acquisition time for the b = 1,000 s/mm^2^ shell was 3 min and 23 s, and for the b = 2,500 s/mm^2^ shell was 4 min and 53 s.

The FMRIB Software Library (FSL) version 6.0.2 (Oxford, United Kingdom) was used for imaging processing and DTI parameter computation per steps previously reported (Owen et al., 2013; Chang et al., 2015; Payabvash et al., 2019). FSL’s topup was used on each diffusion shell to correct for susceptibility induced distortion (Andersson et al., 2003). FSL’s Eddy was used on the diffusion data to correct for motion and eddy distortions, skull stripping, outlier replacement, susceptibility-by-movement, and slice-to-volume correction. FSL’s DTIFIT was used to calculate all DTI parameters: FA, radial diffusivity (RD), axial diffusivity (AD), and mean diffusivity (MD), with maps from the coregistered *b* = 0 s/mm^2^ and *b* = 1,000 s/mm^2^ images. Normalized multi-shell data was used to quantify NODDI parameters: NDI, orientation dispersion index (ODI), and free water fraction (FISO) with the Accelerated Microstructure Imaging *via* Convex Optimization (AMICO) Toolbox (Daducci et al., 2015).

Tract Based Spatial Statistics (TBSS) were performed on DTI and NODDI derived metrics using FSL. The Johns Hopkins University (JHU) ICBM-DTI-81 White-Matter Labeled Atlas and the JHU White-Matter Tractography Atlas, embedded in FSL, were used to extract the average DTI and NODDI values of 20 bilateral and 6 central tracts (total of 46 tracts) in automated fashion. Lateralization Index (LI) was calculated as (Right–Left)/(Right+Left) for each of the 20 bilateral tracts; thus, a left-lateralized tract had a negative LI while a right-lateralized tract had a positive LI.

All statistical analysis was performed using Stata (StataCorp. 2021. *Stata Statistical Software: Release 17*. College Station, TX: StataCorp LLC) and Matlab (Matlab, The MathWorks, Natick, MA, United States). The mean value for each parameter and 95% confidence interval was obtained for the lateralization indices of each of the bilateral tracts. Cohen’s d effect sizes were obtained and a statistical significance threshold of 0.05 was used after Benjamini–Hochberg false discovery rate correction for multiple tract-wise comparisons. Two-tailed heteroscedastic t-tests were run to determine group differences in LI between SOR and non-SOR cohorts with DTI and NODDI metrics. Since this group comparison was an exploratory analysis, multiple comparison correction was not performed.

## Results

### Demographics

Eighty seven subjects were included in the study of which 29 were females and 58 were males; 9 of the 87 subjects were left-handed while 75 were right-handed, handedness data was not available for 3 subjects. Forty eight of the 87 children had SOR while 39 did not have SOR.

### Lateralization index

#### DTI

Please refer to Table 1 caption for white matter tract abbreviations.

**Table 1:** A and B: Cohen’s *D* effect size and *p*-values (*p*-values corrected for multiple comparison) for LI of 20 bilateral tracts for DTI and NODDI metrics.

(A) DTI								
Tract	FA		AD		MD		RD		*p*-value	Cohen’s *D* effect size	*p*-value	Cohen’s *D* effect size	*p*-value	Cohen’s *D*	*p*-value	Cohen’s *D*
ACR	0.878	−0.0233	**<0.001**	0.67	**0.001**	0.512	0.527	0.103
ALIC	**<0.001**	1.01	**<0.001**	2.54	**<0.001**	1.48	0.173	0.217
CGC	**<0.001**	−3.34	**<0.001**	−1.85	**<0.001**	1.54	**<0.001**	3.38
CGH	0.161	0.228	**0.002**	0.499	0.073	0.282	0.811	−0.0364
CP	0.428	0.132	**<0.001**	1.3	**<0.001**	0.923	**0.013**	0.399
CST	**<0.001**	−0.94	**0.011**	0.398	**<0.001**	1.85	**<0.001**	1.77
EC	**<0.001**	−1.38	**<0.001**	1.73	**<0.001**	2.72	**<0.001**	2.56
FXST	**0.002**	−0.495	**<0.001**	1.54	**<0.001**	1.75	**<0.001**	1.19
ICP	**<0.001**	1.39	**0.006**	0.437	**0.002**	−0.489	**<0.001**	−1.11
ML	**<0.001**	−0.865	**0.001**	0.509	**<0.001**	1.04	**<0.001**	1.16
PCR	0.455	0.12	**<0.001**	1.11	**<0.001**	1.4	**<0.001**	0.92
PLIC	**<0.001**	−0.905	<0.001	2.13	**<0.001**	2.2	**<0.001**	1.75
PTR	**<0.001**	−0.756	0.578	0.0848	**<0.001**	0.699	**<0.001**	0.927
RLIC	**<0.001**	−2.43	0.157	0.221	**<0.001**	2.09	**<0.001**	2.94
SCP	**<0.001**	−1.18	**<0.001**	0.698	**<0.001**	1.19	**<0.001**	1.36
SCR	**<0.001**	−1.27	**0.008**	0.42	**<0.001**	1.18	**<0.001**	1.31
SFO	**<0.001**	0.69	**0.001**	0.545	0.788	0.0486	**0.037**	−0.332
SLF	**<0.001**	−1.09	**<0.001**	1.87	**<0.001**	2.6	**<0.001**	1.92
SS	**0.006**	−0.44	**<0.001**	1.42	**<0.001**	2.24	**<0.001**	1.65
UNC	**<0.001**	1.63	**<0.001**	1.48	0.851	0.0285	**<0.001**	−1.13
(B) NODDI								
Tract	NDI				ODI		FISO		*p*-value		Cohen’s *D* effect size		*p*-value	Cohen’s *D* effect size	*p*-value	Cohen’s *D*
ACR	**<0.001**		−1.04		**<0.001**	−0.777	**<0.001**	−0.598
ALIC	**<0.001**		−1.2		**<0.001**	−2.19	**<0.001**	0.544
CGC	**<0.001**		−2.71		**<0.001**	2.5	0.725	−0.0534
CGH	**<0.001**		−1.18		**<0.001**	−0.746	**<0.001**	−0.759
CP	**0.002**		−0.488		**<0.001**	−0.876	**<0.001**	0.635
CST	**<0.001**		−0.589		0.88	−0.0231	**<0.001**	2
EC	**<0.001**		−2.22		**<0.001**	−1.53	**0.013**	−0.402
FXST	**<0.001**		−1.09		**<0.001**	−0.692	**<0.001**	0.547
ICP	**<0.001**		0.58		**<0.001**	−1.33	**0.002**	−0.513
ML	0.127		0.234		0.067	0.293	**<0.001**	1.21
PCR	**<0.001**		−1.19		**0.007**	−0.435	0.415	0.13
PLIC	**<0.001**		−1.19		**<0.001**	−0.925	**<0.001**	1.15
PTR	**0.003**		−0.466		**0.039**	−0.334	0.387	0.143
RLIC	**<0.001**		−1.16		**<0.001**	1.01	**<0.001**	0.813
SCP	**<0.001**		−0.701		0.072	−0.282	**<0.001**	0.931
SCR	**0.004**		−0.454		**0.039**	0.332	**<0.001**	0.87
SFO	**0.001**		−0.549		**<0.001**	−0.647	0.323	−0.167
SLF	**<0.001**		−1.53		0.072	−0.28	**<0.001**	0.966
SS	**<0.001**		−1.12		**<0.001**	−0.985	**0.021**	0.369
UNC	**<0.001**		−0.719		**<0.001**	−2.53	**<0.001**	−1.16

A *p* < 0.05 indicates significant lateralization of the tract. Positive effect size indicates right lateralization while negative effect size indicates left lateralization.

White matter tract abbreviations: *Commissural tracts*: (corticocortical tracts connecting left and right hemispheres): splenium (SCC), body (BCC), and genu (GCC) of the corpus callosum; *Brainstem tracts*: inferior (ICP), middle (MCP), and superior (SCP) cerebellar peduncles, pontine crossing tract (PCT), and medial lemniscus (ML); *Projection tracts*: (cortical to subcortical regions): corticospinal tract (CST), cerebral peduncle (CP), posterior thalamic radiation (PTR), internal capsule (subdivided into anterior limb (ALIC), posterior limb (PLIC), and retrolenticular (RLIC) portions), and corona radiata (subdivided into anterior (ACR), superior (SCR), and posterior (PCR) portions); *Limbic tracts*: cingulum (subdivided into cingulate (CGC) and hippocampal (CGH) portions), fornix (FX), and fornix stria terminalis (FXST); *Association tracts*: (corticocortical tracts within same hemisphere): external capsule (EC), superior fronto-occipital fasciculus (SFO), superior longitudinal fasciculus (SLF), sagittal stratum (SS), and uncinate fasciculus (UNC). The bold values indicate statistically significant *p* values (*p* < 0.05).

**Figure 1:**
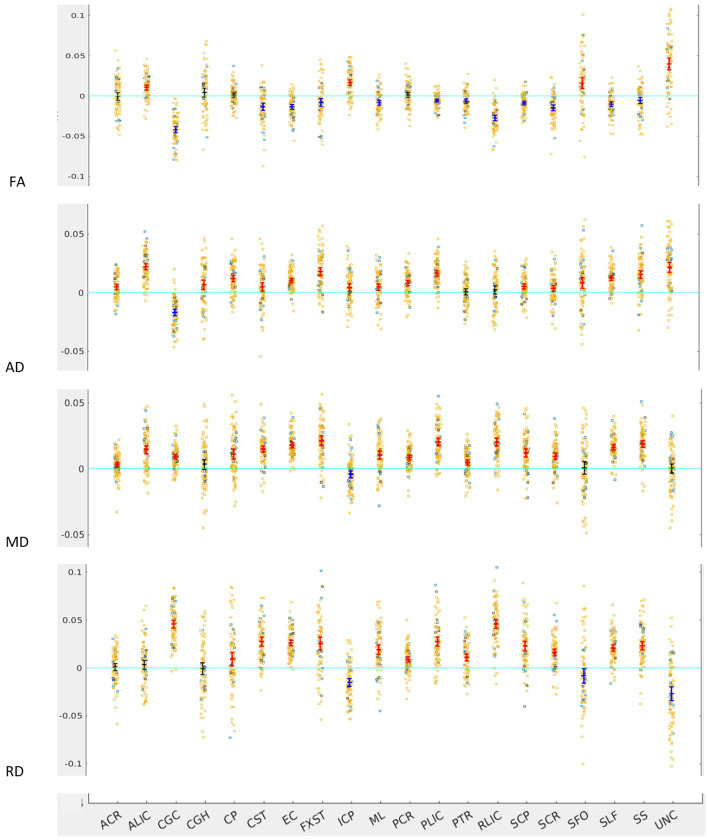
LI plotted for each of the 87 participants for the 20 bilateral tracts for DTI metrics, the mean and 95% CI are represented in the figure, 95% CI bars in red represents significant right lateralization (positive LI) of the tract while blue bar represents significant left lateralization (negative LI) of the tract. Yellow dots represent right-handed subjects (79) while blue dots represent left-handed subjects (9), subjects where handedness data was missing are plotted in black (3).

Across the full cohort, the greatest number of tracts demonstrated hemispheric lateralization for axial diffusivity (AD) with 17 of the 20 bilateral tracts being right lateralized for AD (Figure 1). CGC was strongly left lateralized, as expected from prior studies. PTR and RLIC did not show significant lateralization. For the right lateralized tracts, the Cohen’s D effect sizes ranged from 0.4 to 2.5; for the left lateralized CGC, it was −1.85 (Table 1).

For fractional anisotropy (FA), most (12 of the 20) tracts were left lateralized. ALIC, ICP, SFO and UNC were right lateralized, while ACR, CGH, CP and PCR did not demonstrate any significant lateralization. The left lateralization of FA in most tracts was likely explained by the significant right lateralization of RD in these tracts, which was seen in 14 of 20 regions with Cohen’s D values ranging from 0.4 to 3.4 (Table 1).

#### NODDI

**Figure 2:**
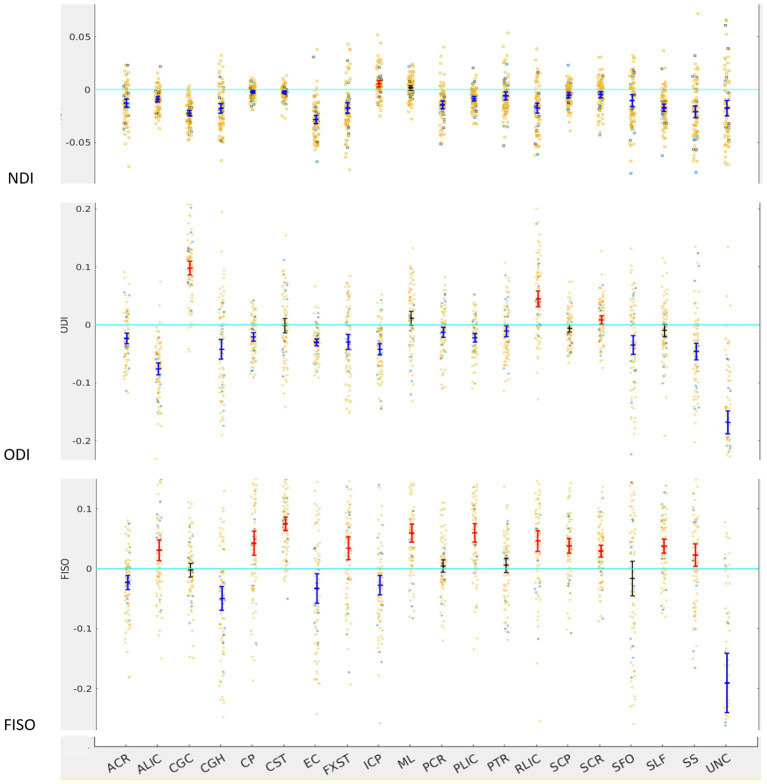
LI plotted for each of the 87 participants for the 20 bilateral tracks for DTI metrics, the mean and 95% CI are represented in the figure, 95% CI bars in red represents significant right lateralization (positive LI) of the tract while blue bar represents significant left lateralizatin (negative LI) of the tract. Yellow dots reperesent right-handed subjects (79) while blue dots represent left-handed subjects (9), subjects where handedness data was missing are plotted in black (3).

For NDI, almost all tracts were left lateralized (18 of the 20) (Figure 2) whereas ICP was right lateralized, and ML did not show any evidence of lateralization. Cohen D effect sizes ranged from 0.5 to 2.7 (Table 1).

Most tracts were left lateralized for ODI (15 of 20 tracts). CGC, RLIC and SCR were right lateralized, whereas CST, ML, SCP and SLF were not significantly lateralized.

Lateralization effects were also seen in the free water fraction (FISO) of 16 of 20 tracts. Most tracts were right lateralized for FISO, whereas ACR, CGH, EC, ML and UNC were left lateralized.

#### SOR vs. non-SOR

**Figure 3:**
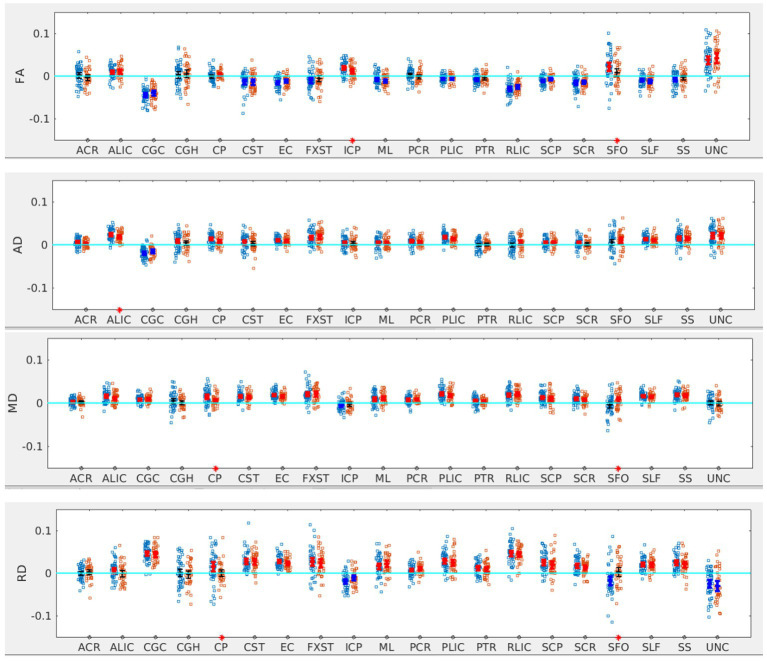
LI plotted for each of the 87 participants for the 20 bilateral tracks for DTI metrics comparing SOR (blue dots) to non-SOR (red dots) means with 95% CI are resented in the figure for SOR and Non SOR, 95% CI bars in red represents significant right lateralization (positive LI) of the tract while blue bar represents significant left lateralizatin (negative LI) of the tract. Red asterisks on the X axis represent tracts for which difference between SOR vs. non SOR was significant at *p* < 0.05.

There was no significant difference in the age, sex distribution, WISC full scale Intelligence Quotient (WISC-FSIQ) scores or the number of left versus right-handed subjects between the SOR and non-SOR groups (Table 2). Overall, there was no significant difference in age, WISC FSIQ scores or left versus right handedness between males and female subjects. Of the 20 bilateral tracts compared using the DTI metrics, the SFO was found to have increased right lateralization for FA and increased left lateralization for MD and RD (*p* < 0.05) in SOR children versus non-SOR children (Figure 3; Table 3). The ICP showed increased rightward lateralization for FA while CP showed increased right lateralization for MD and RD among SOR children.

**Table 2:** Demographic characteristics of children with non-SOR and SOR, continuous variables are reported as mean ± SD while categorical data is represented with absolute numbers, unpaired *t* test is used for continuous data while Pearson chi square test is used for categorical data.

	Non SOR (*N* = 39)	SOR (*N* = 48)	*p*-value
Age	10.3 ± 1.6	10.2 ± 1.7	0.71
Sex			
Male	27	31	
Female	12	17	0.65
Handedness			
Left	2	7	
Right	34	41	0.19
WISC full scale IQ	105.9 ± 12.9	106.1 ± 14.7	0.95

**Table 3:** *p*-values for the significant tracts when comparing SOR to non-SOR for all participants and for males and females separately (see Figures 3–6).

Metric	All participants		Males		Females		Tract	*p*-value	Tract	*p*-value	Tract	*p*-value
FA
	ICP	0.03	ICP	0.02	CGC	0.04
	SFO	0.04	SFO	<0.01	CST	0.03
					SCP	0.04
AD
	ALIC	<0.05	ALIC	0.02	CST	0.02
MD
	CP	0.02	ALIC	0.03		
	SFO	0.02	SFO	0.01		
RD
	CP	0.04	SFO	<0.001	SCR	<0.05
	SFO	<0.01				
ODI
	SCP	0.02			SCP	<0.001
FISO
	CP	<0.01	CP	<0.05	CP	<0.01
	PCR	0.03	PCR	<0.01		
	SFO	0.02	SFO	<0.01		

**Figure 4:**
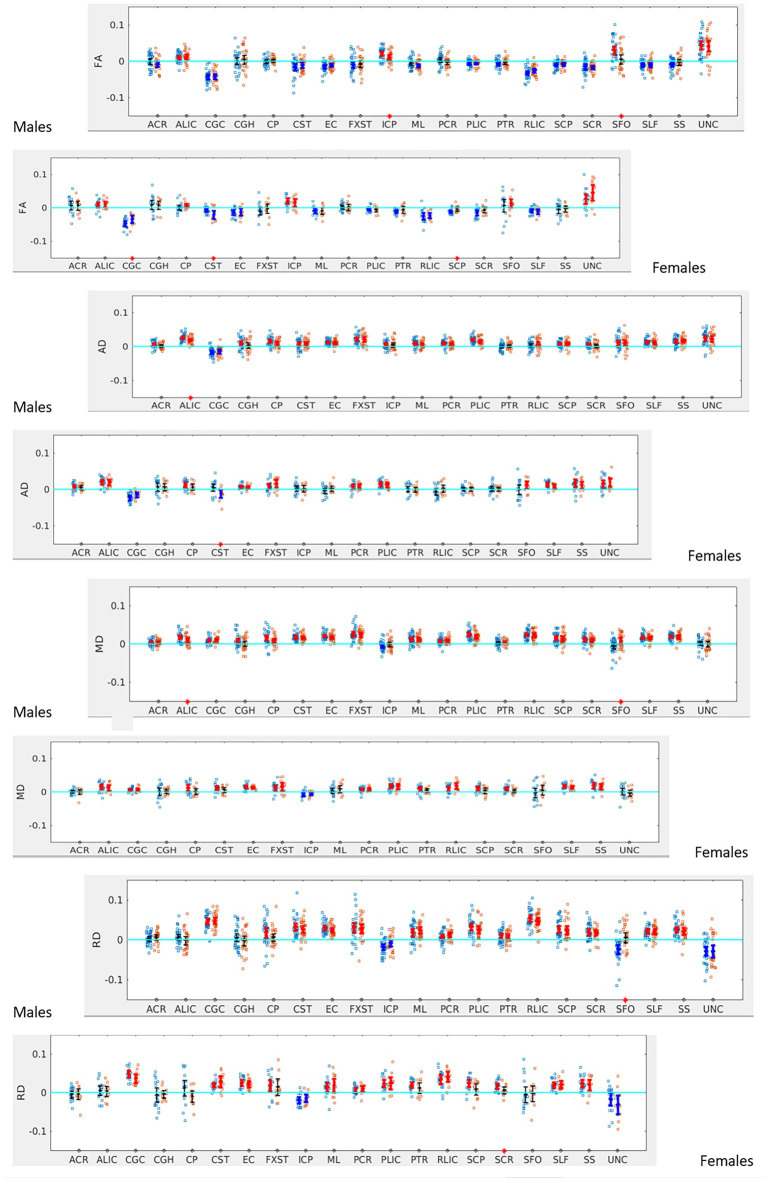
LI plotted for boys (*n* = 58) and girls (*n* = 29) separately for the 20 bilateral tracts for DTI metrics comparing SOR (blue dots) to non-SOR (red dots) means with 95% CI are resented in the figure for SOR and Non SOR, 95% CI bars in red represents significant right lateralization (positive LI) of the tract while blue bar represents significant left lateralizatin (negative LI) of the tract. Red asterisks on the *X* axis represent tracts for which difference between SOR vs. non SOR was significant at *p* < 0.05.

When the males and females were analyzed separately, we found that males with SOR had increased right lateralization in the SFO for FA (*p* = 0.003) and increased left lateralization for MD (*p* = 0.01) and RD (*p* < 0.001) compared to males without SOR (Figure 4; Table 3). Females with SOR showed increased left lateralization of SCP and CGC in FA (p < 0.05) and increased right lateralization of CST in FA and AD and SCR in RD.

**Figure 5:**
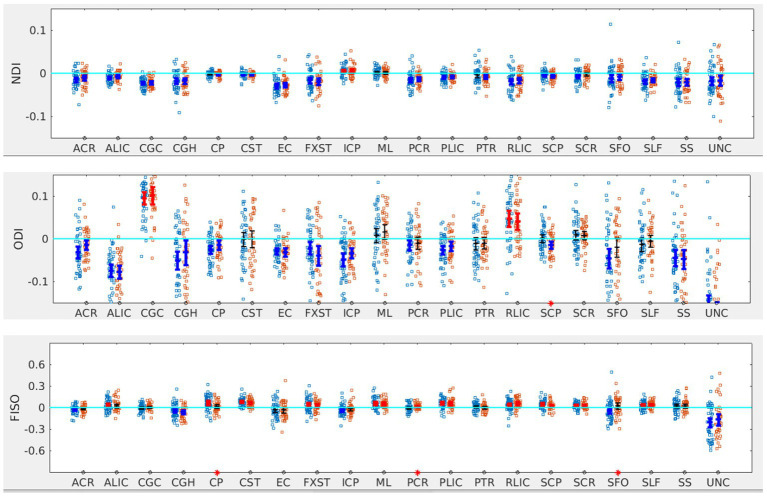
LI plotted for each of the 87 participants for the 20 bilateral tracks for NODDI metrics comparing SOR (blue dots) to non-SOR (red dots) means with 95% CI are resented in the figure for SOR and Non SOR, 95% CI bars in red represents significant right lateralization (positive LI) of the tract while blue bar represents significant left lateralizatin (negative LI) of the tract. Red asterisks on the X axis represent tracts for which difference between SOR vs. non SOR was significant at *p* < 0.05.

In terms of NODDI metrics, the SOR group showed decreased left lateralization of ODI in SCP compared to the non-SOR group (Figure 5). There were differences in free water fraction (FISO) with increased right lateralization for CP and reversal of lateralization for PCR and SFO (Figure 5).

**Figure 6:**
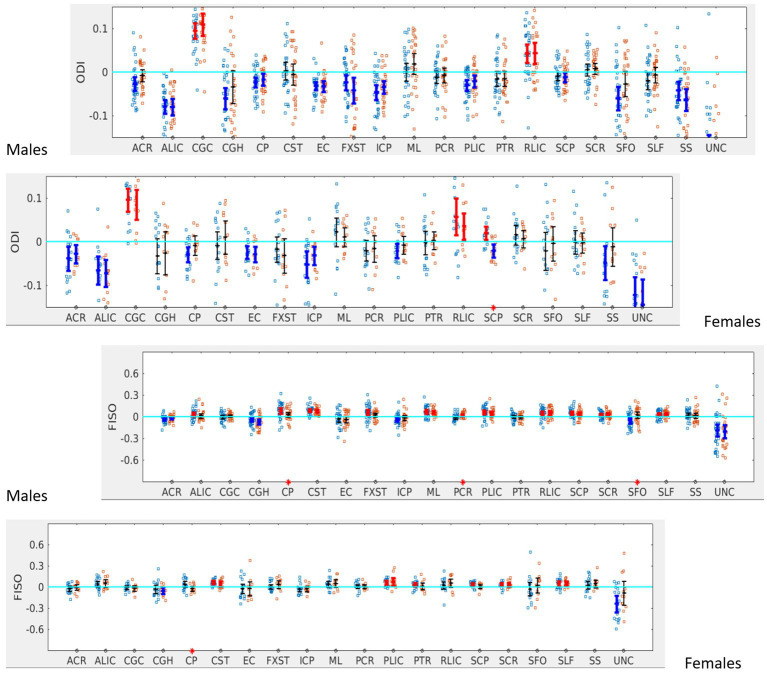
LI plotted for boys (*n* = 58) and girls (*n* = 29) separately for the 20 bilateral tracts for ODI and FISO comparing SOR (blue dots) to non-SOR (red dots) means with 95% CI are resented in the figure for SOR and Non SOR, 95% CI bars in red represents significant right lateralization (positive LI) of the tract while blue bar represents significant left lateralizatin (negative LI) of the tract. There were no significant differences between SOR and non-SOR NDI and hence these are not analyzed saperatly for males and females. Red asterisks on the *X* axis represent tracts for which difference between SOR vs. non SOR was significant at *p* < 0.05.

When the males and females were analyzed separately, females with SOR showed a reversal of lateralization of ODI for SCP compared to females without SOR (*p* < 0.001) (Figure 6; Table 3). Males with SOR showed reversal of lateralization signal for PCR (*p* < 0.01) and SFO (p < 0.01) compared to males with non-SOR (Figure 6; Table 3).

## Discussion

Our results agree with prior studies that demonstrate hemispheric lateralization of white matter microstructure in school-age children using conventional DTI (Trivedi et al., 2009). However, our investigation represents the most comprehensive evaluation to date of the biophysical properties that contribute to this hemispheric asymmetry of white matter microstructure in the human brain using the more advanced NODDI technique. Furthermore, we saw differences in the degree of lateralization of specific tracts for children with SOR compared to those without SOR, suggesting that this neurodevelopmental phenotype may have a “laterality signature.” Lateralization is affected in association, projection and cerebellar tracts in SOR, with evidence for sexual dimorphism of the SOR trait. NODDI advances the understanding of which components of white matter microstructure contribute to hemispheric lateralization in specific tracts and how they are altered in neurodevelopmental disorders.

### Hemispheric asymmetry of white matter microstructure in the human brain

For the overall cohort, most tracts were left lateralized by the FA measure. This is consistent with prior studies showing left lateralization of FA (Trivedi et al., 2009). It can be argued that this may be secondary to most people being right-handed in the population. However, when we separated the right- and left-handed individuals, there was no obvious relationship between lateralization and handedness, in agreement with prior DTI studies (Gong et al., 2005). The findings may be related to functional lateralization of the brain for e.g., speech and language processing tend to occur on the left side. Prior studies have indeed shown loss of left lateralization of FA in large association tracts in autistic adolescents compared to neurotypical children, suggesting that this may be related to impairment of social and communication function located on the left side of the brain (Lo et al., 2011).

The NODDI model allows more specificity regarding microstructural characteristics. The tracts with the greatest left lateralization of FA such as CGC and RLIC combine left lateralization of NDI with right lateralization of ODI, since this reflects higher axonal density and lower fiber orientation dispersion of the left-sided pathway, both of which contribute to more diffusion anisotropy on DTI. Likewise, the tracts with the greatest right lateralization of FA and AD such as the UNC and SFO combine the left lateralization of ODI and FISO, reflecting the higher fiber orientation dispersion on the left side as well as increased free water on the left side, leading to decreased diffusion anisotropy on the left side and consequent right lateralization of diffusion anisotropy in these tracts.

A majority of the tracts (ALIC, CP, CST, ML, PLIC, RLIC, SCP, SCR, SLF, and SS) that demonstrate left lateralization of FA are right lateralized on FISO. These tracts except ML also demonstrate left lateralization of NDI. These findings signify that the left lateralization of FA in these tracts is driven by the increased highly anisotropic intra-axonal water compartment and by the decreased isotropic free water fraction of the left-sided tract compared to the homologous right-sided tract. In the case of ML, there is higher axonal density on the right side as demonstrated by right lateralization of NDI; however, this is outweighed by the higher free water content on the right side leading to left lateralization of FA. Thus, NODDI analysis allows better understanding of the underlying microstructure of different tracts within the human brain and the related findings on standard DTI metrics.

### Hemispheric laterality as a biomarker of sensory over-responsivity?

Our study is the first to evaluate LI in children with SOR and SPD. To our knowledge, we are also the first to characterize in detail the hemispheric lateralization of white matter microstructure using NODDI in children with neurodevelopmental concerns. By contrasting the hemispheres of a single individual, measures of lateralization solve the major problems of the large inter-individual variability of DTI and NODDI metrics as well as the variation produced by different MRI scanner hardware, software, and imaging protocols in multicenter studies and in routine clinical practice.

In terms of SPD, there was a trend toward increased lateralization in SOR children. This contrasts with decreased lateralization reported in studies of autism (Lo et al., 2011). This may be because SOR is distinct from autism (Tavassoli et al., 2018), and different forms of SPD might explain some of the heterogeneity seen in the findings of ASD studies (Tavassoli et al., 2018).

Association tracts like SFO were significantly more lateralized in SOR males, whereas cerebellar (SCP) and projection tracts (CST and SCR) showed altered lateralization in females. We have previously shown altered cerebellar white matter microstructure in children with SPD compared to typically developing children (Narayan et al., 2020). The current study shows alterations in lateralization of cerebellar white matter in females with SPD. This reinforces recent studies which show the cerebellum to be lateralized and cerebral-cerebellar tract lateralization likely secondary to lateralization of brain function (Kang et al., 2015). The sex differences in the tracts affected may reflect differences in the phenotypic manifestation of SPD between males and females (Osório et al., 2021). The distinct signatures in microstructure in males versus females may be secondary to biological differences in SOR in males versus females. Prior studies have shown differences in myelination and cerebral white matter architecture between males and females (Kodiweera et al., 2016; Geeraert et al., 2019; Buyanova and Arsalidou, 2021).

We have also previously shown altered white matter microstructure in the posterior projection tracts in children with the auditory over-responsivity subtype of SOR (Tavassoli et al., 2019). It is likely that, when looking at SOR as a whole, large association tracts may be involved as these may be affected in multisensory integration in children. Our finding of changes in lateralization within the SFO is probably reflective of this.

Analysis of NODDI metrics shows a trend toward increased lateralization among the children with SOR. However, the greatest differences were seen in ODI and FISO, suggesting that differences in microstructure in SOR are likely due to altered fiber orientation dispersion and altered free water content within tracts. A study in adults with autism (Andica et al., 2021) has shown increased FISO and decreased NDI mainly in the commissural and long-range association fibers with predominant distinct sides depending on the tract involved. The study also showed higher specificity and accuracy with NODDI parameters, especially FISO, than standard DTI parameters. In our cohort with SOR, we did find differences in fiber orientation dispersion and free water fraction and there was also a difference in the ODI and FISO patterns for girls versus boys. Prior studies in adults have shown NODDI to have better sex discrimination than standard DTI metrics (Kodiweera et al., 2016). The differences in NODDI parameters are important in understanding the neurobiological basis of distinct phenotypes and further hypothesis-driven studies are warranted in this area to confirm the exploratory findings in this report on hemispheric asymmetry differences in those with SOR versus non-SOR SPD.

### Limitations and future directions

A limitation of the study is that children without SOR are a heterogenous group and a large control group of typically developing children was not available for comparison. The non-SOR children may have various forms of SPD such as sensory under responsivity, sensory craving, and sensory discrimination disorder. These forms of SPD may have different patterns of microstructural changes that can be explored in larger cohorts. In addition, SOR can be divided into subtypes including auditory over-responsivity, visual over-responsivity and tactile over-responsivity as well as mixed subtypes. We did not distinguish the different subtypes in this analysis. Different subtypes may involve different tracts which are involved in the sensory pathway and may account for the differences seen in multiple tracts. These differences in different subtypes of SOR can be explored in larger cohorts. We did not account for puberty which may possibly affect the brain imaging and phenotypic results. Our cohort was aged 8–12. Even though there has been a trend toward earlier puberty in girls, given the narrow range and the young age of the study group, children in this study are less likely to have undergone puberty (Brix et al., 2019).

As mentioned in the methods section since the group comparison was an exploratory analysis multiple comparison correction wasn’t performed. However, these results are hypotheses generating and can be the bases for studies in larger cohorts to investigate these differences in detail.

## Conclusion

Our study shows that advanced dMRI provides valuable insights into the neurobiological basis of sensory processing dysfunction, which is now recognized as a fundamental feature of ASD as well as a significant neurodevelopmental challenge for many children not on the autistic spectrum. SOR shows distinctive laterality differences from non-SOR SPD in white matter microstructure which differs between males and females. Assessing hemispheric asymmetry may help eliminate the variability associated with scanner hardware, software, scan protocols and also interindividual variation. Hence, dMRI measures of lateralization may potentially serve as clinically useful imaging biomarkers for neurodevelopmental disorders such as sensory processing dysfunction, including all its subtypes. Better elucidating the white matter microstructure and connectivity of those with SPD might also aid neuroscientific research on sensory processing, which is the early developing gateway into the brain, and which needs to develop normally for downstream systems such as language and attention to then develop and function properly.

## Data availability statement

The datasets presented in this study can be found in online repositories. The names of the repository/repositories and accession number(s) can be found at: The raw data have been submitted to the NIMH Data Archive (NDA) for public dissemination.

## Ethics statement

The studies involving human participants were reviewed and approved by UCSF Committee on Human Research institutional review board. Written informed consent to participate in this study was provided by the participants’ legal guardian/next of kin.

## Author contributions

SP carried out image processing, and analyses interpreted the findings, wrote, and revised the manuscript. JW-J, IB, and LC assisted in data collection, analysis, and in interpretation of findings and revised the manuscript. ML, MR, RC, KT, and RG performed the SP3D assessments and collected and assisted in the interpretation of data, and revised the manuscript. PM and EM designed the study, interpreted findings, and contributed to the writing and revision of the manuscript. All authors contributed to the article and approved the submitted version.

## Funding

The study was funded by the National Institute of Health R01 grant, award number MH116950. The research was conducted in the absence of any commercial or financial relationships that could be construed as a potential conflict of interest.

## Conflict of interest

The authors declare that the research was conducted in the absence of any commercial or financial relationships that could be construed as a potential conflict of interest.

## Publisher’s note

All claims expressed in this article are solely those of the authors and do not necessarily represent those of their affiliated organizations, or those of the publisher, the editors and the reviewers. Any product that may be evaluated in this article, or claim that may be made by its manufacturer, is not guaranteed or endorsed by the publisher.
